# Phylogeny of certain members of Hyrcanus group (Diptera: Culicidae) in China based on mitochondrial genome fragments

**DOI:** 10.1186/s40249-019-0601-1

**Published:** 2019-10-23

**Authors:** Hui-Min Zhu, Shu-Han Luo, Man Gao, Feng Tao, Jing-Peng Gao, Han-Ming Chen, Xiang-Yu Li, Heng Peng, Ya-Jun Ma

**Affiliations:** 1 College of Basic Medical Sciences, Second Military Medical University, Shanghai, 200433 China; 2 Department of Naval Medicine, Second Military Medical University, Shanghai, 200433 China; 3 Department of Medical Microbiology and Parasitology, Second Military Medical University, Shanghai, 200433 China

**Keywords:** *Anopheles hyrcanus* group, Mitochondrial genome fragment, Phylogenetic relationship

## Abstract

**Background:**

Species of the *Anopheles hyrcanus* group are widely distributed in Palearctic and Oriental regions and some of them are important malaria vectors. The cryptic species of *An. hyrcanus* group was almost impossible to identify based only on their morphology. The phylogenetic relationship of *An. hyrcanus* group was also not clear.

**Methods:**

Five members of *An. hyrcanus* group were identified by rDNA ITS2 sequencing as *An. yatsushiroensis, An. belenrae, An. kleini, An. lesteri* and *An. sineroides.* The mitochondrial genome fragments were sequenced and annotated using the mitochondrial genome of *An. sinensis* as reference. Based on the four segments and Joint Data sequences of these species, and other four anopheline species downloaded from GenBank, intraspecific as well as interspecific genetic distances were calculated and the phylogenetic trees were reconstructed by the methods of neighbor joining, maximum parsimony, minimum evolution and maximum likelihood.

**Findings:**

Four parts of mitochondrial genomes, which were partial fragments *COI + tRNA + COII* (F5), *ATP6 + COIII*(F7 + F8), *ND1*(F19) and *lrRNA* (F21), were obtained. All fragments were connected as one sequence (referred as Joint Data), which had a total length of 3393 bp. All fragment sequences were highly conservative within species, with the maximum *p* distance (0.026) calculated by F19 of *An. belenrae*. The pairwise interspecific *p* distance calculated by each fragment showed minor or even no difference among *An. sinensis*, *An. kleini* and *An. belenrae*. However, interspecific *p* distances calculated by the Joint Data sequence ranged from 0.004 (*An. belenrae* vs *An. kleini*) to 0.089 (*An. sineroides* vs *An. minimus*), and the *p* distances of the six members of *An. hyrcanus* group were all less than 0.029. The phylogenetic tree showed two major clades: all subgenus *Anopheles* species (including six members of *An. hyrcanus* group, *An. atroparvus* and *An. quadrimaculatus* A) and subgenus *Cellia* (including *An. dirus* and *An. minimus*). The *An. hyrcanus* group was divided into two clusters as ((*An. lesteri, An. sineroides*) *An. yatsushiroensis*) and ((*An. belenrae, An. sinensis*) *An. kleini*)).

**Conclusions:**

The *An. hyrcanus* group in this study could be divided into two clusters, in one of which *An. belenrae, An. sinensis* and *An. kleini* were most closely related. More molecular markers would make greater contribution to phylogenetic analysis.

## Multilingual abstracts

Please see Additional file 1 for translations of the abstract into the five official working languages of the United Nations.

## Background

*Anopheles hyrcanus* group belongs to the subgenus *Anopheles*, genus *Anopheles*. It is widely distributed in Palearctic and Oriental regions, including 25 species with valid reported mosquito species [1]. There were 22 species of *An. hyrcanus* group distributed in China, including three unnamed ones [2]. Some cryptic species of *An. hyrcanus* group have similar morphological characteristics, making it difficult to identify them based only on their morphology [3]. Moreover, quite a few hybridized individuals were found in field [4, 5] and reestablished phylogenetic trees of *An. hyrcanus* group showed disparity according to various molecular markers [3, 6, 7]. These facts illustrated the complex phylogenetic relationships within *An. hyrcanus* group.

Mitochondrial genome strictly followed maternal inheritance in structure as well as in evolution, with abundant information for genetic and phylogenetic population studies. The mitochondrial genome of *Anopheles* mosquitoes consisted of 13 protein-coding genes, 22 transfer RNA (*tRNA*) genes, two ribosomal RNA (*rRNA*) genes and an AT-rich control region [8–10]. At present, certain genes of mitochondrial genome were employed to analyze the interspecific or intraspecific differences. For instance, *COI* sequence was used as DNA barcoding to distinguish mosquito species [11, 12], while genes such as *COI* [13], *COII* [14], *ND5* [15] and control region [16] were utilized to detect the genetic population structure of Hyrcanus group members.

So far, we have reported the complete mitochondrial genome of *An. sinensis* in Hyrcanus group [9]. In this study, we sequenced and analyzed mitochondrial genome fragments of *An. hyrcanus* group members in China in order to reestablish the phylogenetic relationships and determine the taxonomic status of cryptic species. Moreover, we discussed the contribution of mitochondrial genome fragments in phylogenetic study.

## Materials and methods

### Ethics statement

No permits were required for the described field studies. Adult mosquito collection in chicken farms and livestock pens was agreed by the owners at each location.

### Mosquito collection and species identification

With the consent of the owners, mini light traps (MYFS-HJY-1, Houji Shenzhen, China) were set up in chicken farms and livestock pens between 6:30 pm and 7:30 am. Then the captured *Anopheles* specimens were collected by the mini light traps and manually by entomological aspirators in the evening and killed by freezing before being individually transferred to laboratory in centrifuge tubes for further analysis (Table 1). In the light of the taxonomic key by Lu et al. [17], the samples were morphologically identified as members of *An. hyrcanus* group.

**Table 1:** Information collected from the members of *Anopheles hyrcanus* group in China

Species	Collection sites	Date	Number of samples
*Anopheles yatsushiroensis*	Wuxiang, Shanxi	June 2017	8
	Taian, Shandong	July 2017	3
	Xingcheng, Liaoning	August 2008	2
*An. belenrae*	Jining, Shandong	July 2017	2
	Donggang, Liaoning	July 2018	2
*An. kleini*	Wuxiang, Shanxi	June 2017	11
	Tongliao, Inner Mongolia	August 2018	3
*An. lesteri*	Jiangsu, Lab colony		6
*An. sineroides*	Kuandian, Liaoning	August 2018	1

The member species of *An. hyrcanus* group was further identified by molecular markers with rDNA ITS2 sequences. Single mosquito genomic DNA was extracted using DNAzol (Life Technologies, USA) following the manufacturer’s instructions. DNA pellet was dissolved in 80 μl H_2_O. The rDNA ITS2 fragment was amplified according to the method by Lin et al. and Ma et al. [18, 19]. An ABI 3730 (Boshang Biotech Co., Ltd. Shanghai, China) was applied to purify and sequence the PCR products. Finally, the sequences were Blast aligned in Genebank on the NCBI website to determine the species [18, 19].

### Amplification and sequencing of mitochondrial genome fragments

Some fragments of the mitochondrial genome were amplified referring to the universal primers designed for mitochondrial genome of Diptera [20] (Table 2, Additional file 7: Figure S1). The length range of the amplified product was from 500 bp to 1200 bp and the overlapping length between adjacent sequences was between 50 bp and 485 bp. The range of percentage of GC was from 40 to 60%, while the annealing temperature was either 45 °C to 47 °C. PCR reaction was run in a 25 μl mixture containing 1 μl DNA template, 0.2 μmol/L primers and other PCR reagents (Aidlab Biotechnologies, China). PCR thermal cycling included a 2 min initial denaturation at 94 °C, followed by 30–35 cycles of denaturation at 94 °C for 30 s, annealing at 45 °C/47 °C for 30 s, elongation at 72 °C for 1 min, and a final extension for 8 min at 72 °C. The PCR products were purified and sequenced by an ABI 3730 machine.

**Table 2:** Primers for amplification of mitochondrial genome fragments

Amplication fragments	Primer name	Sequence (5′ → 3′)	Annealing temperature (°C)
F5: *COI* + *tRNA* + *COII*	5-F2637	AGCAGGWTTTRTYCAYTGAT	45
	5-R3590	CTCCTAAAGCWGGKAYTGTT	
F7: *ATP6* + *COIII*	7-F4076	ATTTTCYGTATTYGACCCYTC	47
	7-R4929	TCTCGWGAWACATCTCGTCAT	
F8: *ATP6* + *COIII*	8-F4518	CGACCWGGAACWTTAGCWGT	47
	8-R5523	TAYCCTCCTCATCARTAAAT	
F19: *ND1*	19-F11982	AAAGCAAAWCCYCCTCTTC	47
	19-R12558	ATATTCAAATTCGTAARGG	
F21: *lrRNA*	21-F12834	TTACRCCGGTTTGAACTCAG	47
	21-R13356	WTAAAGTCTAACCTGCCCAC	

### Phylogenetic analysis

The sequences were compared using DNAstar 7.1 (https://www.dnastar.com/software/lasergene/) [21] and annotation as well as splicing was completed referring to the mitochondrial genome of *An. sinensis* (GenBank accession No. KT218684.1) [9]. The intraspecific differences (*p* distance) were calculated by MEGA 7.0 (https://mega.software.informer.com/7.0/) [22] before further analysis was conducted using consensus sequence as the species-specific identity.

The complete mitochondrial genome of *An. sinensis* (KT218684.1), *An. dirus A* (JX219731.1), *An. atroparvus* (KT382817.1), *An. quadrimaculatus* (AL04272.1) and *An. minimus* (KT895423.1) were downloaded from GenBank database. Mitochondrial genome fragment sequences from a total of 10 species were aligned using MEGA 7.0, including *An. yatsushiroensis, An. belenrae, An. kleini, An. lesteri* and *An. sineroides*, in addition to the aforementioned five species downloaded from GenBank. The variable bases, parsimony information bases and nucleotide composition were analyzed by MEGA 7.0. In light of the Joint Data (JD) and the separated segments (F5 (*COI + tRNA + COII*), F7 + F8 (*ATP6 + COIII*), F19 (*ND1*) and F21 (*lrRNA*)), phylogenetic trees were constructed by the methods of neighbor joining (NJ), maximum parsimony (MP) and minimum evolution (ME), respectively. On the other hand, maximum likelihood (ML) tree was reconstructed by PhyML 3.0 (http://phylogeny.lirmm.fr/phylo_cgi/one_task.cgi?task_type=phyml) [23]. The best-fit nucleotide substitution model was obtained by Modeltest 3.7 (http://evomics.org/resources/software/molecular-evolution-software/modeltest/) [24] before ML tree construction. Moreover, bootstrap values for 1000 replicates of all trees were calculated. A congruence length test for JD was performed before analysis [25].

## Results and discussion

### Sequence characters of mitochondrial genome fragments

Five mitochondrial genome fragments out of the five members in *An. hyrcanus* group were obtained. Fragment F5 (893 bp in length) comprised segmental *COI*, full-length *tRNA-Leu* plus segmental *COII* (MK690504–MK690508). Due to a partial overlapping found in Fragment F7 and F8, they were connected (denoted as F7 + F8) for further analysis. Its length was 1407 bp and included segmental *ATP6* and segmental *COIII* (MK825734–MK825738). F19, 583 bp in length, was segmental *ND1* (MK825739–MK825748). F21 was segmental 16 *lrRNA* (MK825744–MK825748) with 510 bp in length. As the combined sequence of all fragments, JD had an aggregate length of 3393 bp.

### Intraspecific differences

The five fragments’ sequences of mitochondrial genome were aligned among the five members of *An. hyrcanus* group in this study. The averages of the nucleotide composition as well as the numbers of conserved and variable bases are shown in Additional file 2: Table S1. All fragments’ sequences were highly conservative within species, with the maximum *p* distance (0.026) calculated by F19 of *An. belenrae* (Table 3). The reported intraspecific differences of mitochondrial genomes in *An. hyrcanus* group were as follows: *An. lesteri* (*COII*: h = 0.000–0.005; *Cyt B*: h = 0.000–0.005) [14], *An. sinensis* (*COI*: *p* = 0.0088, Pi = 0.0039–0.0105; *COII*: *p* = 0.0047; control region: h = 0.00453–0.01617) [13, 16, 26]. The maximum intraspecific distance of *COI* in 17 members of Hyrcanus group was 0.008 (range: 0.002–0.017) [7]. The results indicated varied intraspecific differences among the fragments, suggesting that prudence would be required in the analysis of interspecies relationships within *An. hyrcanus* group. The differences between *COI* sequences increased in higher taxonomic categories [27], while the *COI* barcoding gap was usually 2% within species [28]. High divergence of intraspecific distance was probably caused by recent geographic isolation, indicating the presence of cryptic species [27].

**Table 3:** Intraspecific *p* distances of mitochondrial genome fragments of *Anopheles hyrcanus* group members

Species	Fragments of mitochondrial genome				
	F5	F7	F8	F19	F21
*An.belenrae*	0.008 (0.003–0.013)	N	0.004 (0.000–0.006)	0.018 (0.010–0.026)	0.004
*An. kleini*	0.008 (0.002–0.009)	0.015	0.002 (0.001–0.003)	0.000	N
*An. lesteri*	0.000	0.003 (0.000–0.005)	0.000	0.012	0.000
*An. yatsushiroensis*	0.010	N	0.009 (0.008–0.011)	N	N

The mean precedes the range presented in parentheses. N denotes the absence of data

### Interspecific differences

The four different mitochondrial genome fragments and the JD sequences of the five members of *An. hyrcanus* group in this study, together with the five anopheline species downloaded from the GenBank were analyzed using MEGA 7.0. The ranges of the variable and parsimony information bases were from 46 (F21) to 636 (JD) and 18 (F21) to 363 (JD), respectively, and the averages of GC content varied from 24.6 to 27.4% (Table 4).

**Table 4:** The alignment information of the mitochondrial genome fragments among the 10 anopheline species in this study

Fragment of the mitochondrial genome	Length (bp)	Variable bases (bp)	Parsimony information bases (bp)	Nucleotide composition (%)			
				T	C	A	G
F5 (*COI + tRNA + COII*)	893	182	118	39.1	14.8	34.8	11.3
F7 + 8 (*ATP6 + COIII*)	1407	304	164	32.4	12.4	40.3	15.0
F19 (*ND1*)	583	100	57	47.3	9.6	28.1	15.0
F21(*lrRNA*)	510	46	18	39.0	9.4	34.9	16.7
JD (Joint Data)	3393	636	363	37.7	12.1	36.0	14.3

The pairwise interspecific *p* distance based on the four fragments (F5, F7 + F8, F19, F21) of mitochondrial genome showed that F21 sequence was completely conserved between *An. kleini* and *An. belenrae*, suggesting that F21 fragment had no interspecific resolution. Moreover, other fragments showed minor difference among *An. sinensis*, *An. kleini* and *An. belenrae*. Meanwhile, the *p* distance between subgenus *Cellia* and *Anopheles* species was greater than that among the species within the same subgenus (Additional file 3: Table S2, Additional file 4: Table S3, Additional file 5: Table S4 and Additional file 6: Table S5).

The interspecific *p* distances calculated by the JD sequence ranged from 0.004 (*An. belenrae* vs *An. kleini*) to 0.089 (*An. sineroides* vs *An. minimus*), and the *p* distances of the 6 members of *An. hyrcanus* group were all less than 0.029 (Table 5).

**Table 5:** The pairwise interspecific *p* distances of mtDNA fragments of the 10 mosquito species calculated by Joint Data

	YAT	BEL	KLE	LES	SINE	SIN	DIR	ATR	QUA
BEL	0.020								
KLE	0.020	0.004							
LES	0.026	0.025	0.023						
SINE	0.026	0.028	0.025	0.024					
SIN	0.020	0.005	0.005	0.027	0.029				
DIR	0.076	0.074	0.076	0.081	0.085	0.073			
ATR	0.078	0.074	0.075	0.081	0.082	0.074	0.090		
QUA	0.074	0.071	0.071	0.074	0.078	0.070	0.086	0.063	
MIN	0.083	0.078	0.079	0.082	0.089	0.078	0.084	0.085	0.082

*YAT An. yatsushiroensis*, *BEL An. belenrae*, *KLE An. kleini*, *LES An. lesteri*, *SINE An. sineroides*, *SIN An. sinensis*, *DIR An. dirus A*, *ATR An. atroparvus*, *QUA An. quadrimaculatus*, *MIN An. minimus*

*COI* gene was the most common mitochondrial genome fragment for mosquito identification and genetic relationship analysis [7, 12, 13, 29–34]. In addition, the vast majority of intraspecific distances in 122 mosquito species (15 genera) were at levels from 6 to 15% [12]. On the basis of mtDNA *COI* sequence, the average intraspecific K2P distance of Hyrcanus group 17 species was 0.008 (range: 0.002–0.017) [7]. However, compared with the intraspecific distance, the interspecific distance among cryptic species was smaller, such as *An. sinensis* vs. *An. belenrae* (0.009) [7], *An. liangshanensis* vs *An. kunmingensis* (0.002), and *An. yatsushiroensis* vs *An. junlianensis* (0.003) [35]. The F21 fragments were even the same in both *An. kleini* and *An. belenrae*. Therefore, only partial mitochondrial genome fragment sequences were insufficient to accurately explain the phylogenetic relationship within *An. hyrcanus* group, especially among cryptic species.

### Phylogenetic analysis

**Fig. 1:**
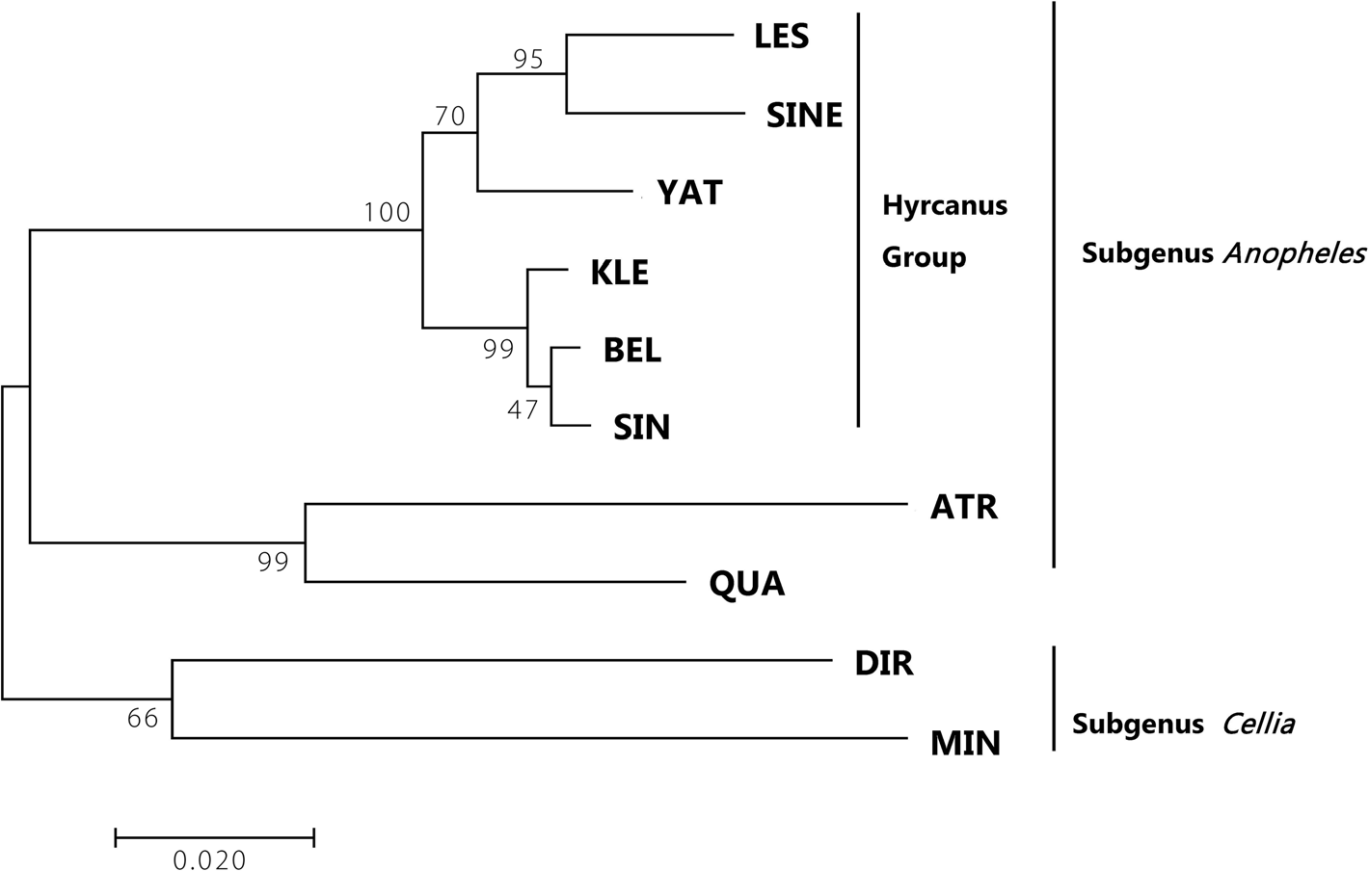
The phylogenetic maximum likelihood tree based on the Joint Data of mitochondrial genome fragments. The codes are the same as those in Table 5, and numbers on the clades denote bootstrap values

The phylogenetic trees were reconstructed using four methods based on the three independent fragments (except F21). All topological structures of the phylogenetic trees based on F7 + F8 and F19 were consistent and split up into two clades. One clade consisted of *An. dirus* and *An. hyrcanus* group six species, while the other included the rest three species (Additional file 8: Figure S2). The topology based on F5 and JD was also consistent. There were two major clades, one of which included all nine species (*An. hyrcanus* group six species, *An. atroparvus* and *An. quadrimaculatus* A) that belonged to subgenus *Anopheles*, and the other contained *An. dirus* and *An. minimus* that belonged to subgenus *Cellia* (Fig. 1). The 6 species of *An. hyrcanus* group were divided into two clusters: ((*An. lesteri, An. sineroides*) *An. yatsushiroensis*)) and ((*An. belenrae, An. sinensis*) *An. kleini*)). The optimal nucleotide substitution models for F5 and JD were *GTR + G* and *GTR + G + I*, respectively. The test result of congruence length for JD showed there was not a congruence length data *(P* = 0.01). The bootstrap values of ML tree were almost above 47% (Fig. 1).

In *An. hyrcanus* group, *An. sinensis* had a very close genetic relationship with *An. belenrae* and *An. kleini*. Their adults shared such similar morphology that there were not enough taxonomic characteristics to distinguish them. *Anopheles belenrae* and *An. kleini* were reported in 2005 [36], and natural hybridization between *An. sinensis* and *An. kleini* were found later [4, 5], indicating possible gene introgression in sympatric population and incomplete reproductive isolation between these two species as well as ongoing speciation. It was known that *rDNA ITS2* sequences were the ideal molecular marker to distinguish cryptic species. However, the length of *rDNA ITS2* in different mosquito species varied greatly, which led to alignment difficultly. Therefore, it was impossible to reconstruct different mosquito species simultaneously. Partial sequences of mitochondrial genome fragments were not able to provide adequate resolution for cryptic species. However, if mitochondrial genome sequences could be sufficiently informative, such as the employment of joint data connection, the phylogenetic tree of cryptic and distant genetic species could be reconstructed simultaneously.

## Conclusions

*Anopheles hyrcanus* group is widely distributed in Palearctic and Oriental regions and some of them are important local malaria vectors. The cryptic species of *An. hyrcanus* group was almost impossible to identify based only on their morphology. In this study, the phylogenetic tree was established by creating mitochondrial genome fragments sequences (JD). It had two major clades, one of which included all Subgenus *Anopheles* species (*An. hyrcanus* group six species, *An. atroparvus* and *An. quadrimaculatus* A) and the other was comprised of *An. dirus* and *An. minimus* that belonged to subgenus *Cellia. Anopheles hyrcanus* group was divided into two clusters as ((*An. lesteri, An. sineroides*) *An. yatsushiroensis*) and ((*An. belenrae, An. sinensis*) *An. kleini*)). More molecular markers would make greater contribution to phylogenetic analysis.

## Supplementary information

**Additional file 1.** Multilingual abstracts in the five official working languages of the United Nations.

**Additional file 2: Table S1.** The mean of nucleotide composition and the numbers of conserved and variable bases of mitochondrial genome fragments in *Anopheles hyrcanus* group 5 members.

**Additional file 3: Table S2.** The pairwise *p* distance between Subgenus *Cellia* and *Anopheles* species in this study calculated by F5 sequences.)

**Additional file 4: Table S3.** The pairwise *p* distance between Subgenus *Cellia* and *Anopheles* species in this study calculated by F7 + F8 sequences.

**Additional file 5: Table S4.** The pairwise *p* distance between Subgenus *Cellia* and *Anopheles* species in this study calculated by F19 sequences. (DOCX 15 kb)

**Additional file 6: Table S5.** The pairwise *p* distance between Subgenus *Cellia* and *Anopheles* species in this study calculated by F21 sequences.

**Additional file 7: Figure S1.** Primers for amplifying mitochondrial genome fragments of Anopheles.

**Additional file 8: Figure S2.** The phylogenetic ML tree reconstructed based on F7 + F8 fragment of mitochondrial genome. YAT: *An. yatsushiroensis*; BEL: *An. belenrae*; KLE: *An. kleini*; LES: *An. lesteri*; SINE: *An. sineroides*; SIN: *An. sinensis*; DIR: *An. dirus A*; ATR: *An. atroparvus*; QUA: *An. quadrimaculatus*; MIN: *An. minimus*. The numbers on the clades denote the bootstrap confidence values.

## Data Availability

The datasets used and/or analyzed during the current study are available from the corresponding authors on reasonable request, and sequences are available in GenBank.

## Abbreviations

*ATP6*: ATPase6
*COI*: Cytochrom oxidase subunit I
*COII*: Cytochrom oxidase subunit II
*COIII*: Cytochrom oxidase subunit III
*Cyt B*: Cytochrom B
*ITS2*: Second internal transcribed spacer
JD: Joint Data
*lrRNA*: Large Rrna
ME: Minimum evolution
ML: Maximum likelihood
MP: Maximum parsimony
*ND1*: NADH dehydrogenase 1
*ND5*: NADH dehydrogenase 5
NJ: Neighbor joining
*rDNA*: Ribosomal DNA
*rRNA*: Ribosomal RNA
*tRNA*: Transfer RNA
