# Supplementary material for: Phylogeny of certain members of Hyrcanus group (Diptera: Culicidae) in China based on mitochondrial genome fragments
Source: Infect Dis Poverty. 2019 Oct 23;8:91. doi: 10.1186/s40249-019-0601-1 (PMC6806543; doi:10.1186/s40249-019-0601-1)
Supplement: Supplementary file 1 — Additional file 1. Multilingual abstracts in the five official working languages of the United Nations. [file 40249_2019_601_MOESM1_ESM.pdf]

Translation of the abstract into the five official working languages of the United Nations

سلالة أعضاء معينين من بعوض هيركانوس (Culicida Duptira:) في الصين استنادًا إلى شظايا جينوم الميتوكوندريا

هيو مين زو، شو هان ليو، مان غاو، فانج تاو، جينغ بينغ غاو، هان مينغ تشن، زانغ يو لي، هانغ بينغ، يا جون ما

نبذة مختصرة

الخلفية: فصيلة بعوض الأنوفيليس مجموعة الهيركانوس تتوزع بشكل واسع في المناطق الشرقية القديمة وبعضها من نواقل الملاريا المهمة.

مجموعة أنوفيليس هيركانوس مبهمة كان من المستحيل تقريبًا تحديدها اعتمادًا على تكوينها. كذلك كانت العلاقة التطورية الجينية لمجموعة الأنوفيليس هيركانوس غير واضحة.

الأساليب: خمسة أعضاء من مجموعة أنوفيليس هيركانوس تم تحديدها بـ(rDNA ITS2) تسلسل أنوفيليس yatsushiroensis، أنوفلبس kleini، أنوفيليس Lester، أنوفليس sineroides. تم تسلسل شظايا جينوم الميتوكوندريا وتعليقها باستخدام جينوم الميتوكوندريا سينينسيس كمرجع. استنادًا إلى الأجزاء الأربعة وتسلسل البيانات المشتركة لهذه الأنواع والأنواع الأربعة الأخرى من الأنوفيلين التي تم تنزيلها من بنك الجينات، تم حساب المسافات الجينية بين الأنواع المحددة، وكذلك ضمن الأنواع المحددة وثم تم إعادة بناء الأشجار الوراثية عن طريق ضم الجوار، الحد الأقصى من الاقتصاد، الحد الأدنى من التطور مع أقصى الاحتمالات.

النتائج: أربعة أجزاء من جينومات الميتوكوندريا والتي كانت شظايا جزئية (COI+tRNA+COII(F5)، ATP6+COIII(F7+F8)، NDI(F19) وlrRNA(F21) استحصلت وتم ربط جميع الأجزاء كسلسلة واحدة يشار إليها باسم (البيانات المشتركة) والتي يبلغ إجمالي طولها 3393 نقطة أساس. 、【كانت جميع متواليات الأجزاء شديدة التحفظ داخل الأنواع مع أقصى مسافة (0.026) لحساب F19 لأنوفيليس belenrae. وجد أن المسافة بين كل زوج من الأنواع محسوب حسب كل جزء طفيفة أو لا فرق بين أنوفيليس sinensis وأنوفيليس kleini وأنوفيليس belenrae. ومع ذلك تراوحت المسافات المحسوبة حسب تسلسل البيانات المشتركة بين 0.004 لأنوفيليس belenrae مقابل أنوفيليس kleini إلى 0.089 أنوفيليس sineroides مقابل أنوفيليس minimus. والمسافات بين الأعضاء الستة لمجموعة الأنوفيليس Hyrcanus كانت أقل من 0.029.

أظهرت شجرة التكاثر الجيني نوعين من الواجهات الرئيسية. واجهة تضم فصيلة أنوفيليس من ضمنها ستة أعضاء من (أنوفيليس Hyrcanus، أنوفيليس atroparvus، وأنوفيليس quadrimaculatus A) وواجهة Cellia (وتتضمن أنوفيليس dirus وأنوفيليس minimus). مجموعة أنوفيليس Hyrcanus قسمت إلى كتلتين (أنوفيليس lesteri، أنوفيليس sineroides، أنوفيليس yatsushiroensis، وأنوفيليس belenrae، أنوفيليس sinensis، أنوفيليس kleini).

الاستنتاجات: مجموعة أنوفيليس hyrcanus في هذه الدراسة يمكن تقسيمها إلى كتلتين، تكون في إحداها أنوفيليس belenrae أنوفيليس sinensis وأنوفيليس kleini مرتبطة مع بعضها بشكل وثيق. المزيد من العلامات الجزيئية من شأنها المساهمة أكثر في التحليل الجيني الوراثي.

Translated from English version into Arabic by Afef Kadi, revised by sjaatoul, through

TRANSLATORS
WITHOUT BORDERS

基于线粒体基因组片段的中国赫坎按蚊种团（双翅目：蚊科）部分成员种的系统发育关系研究

朱慧敏，骆书涵，高曼，陶峰，高景鹏，陈翰明，李翔宇，彭恒，马雅军

摘要

引言：赫坎按蚊种团的成员种广泛分布于古北界和东洋界，其中有些是重要的疟疾传播媒介。仅

根据形态学特征，几乎无法鉴定赫坎按蚊种团中的隐种。同时，赫坎按蚊种团成员种的系统发育关系也尚不完全明确。
方法：通过测定 rDNA ITS2 序列确定赫坎按蚊种团的 5 个成员种，包括：八代按蚊、比伦按蚊、克莱按蚊、雷氏按蚊和类中华按蚊。以中华按蚊的线粒体基因组作为参照，对上述 5 成员种的线粒体基因组片段进行测序和注释，同时在 GenBank 下载了其他 4 种按蚊的对应序列，分析和计算线粒体基因组各独立片段序列，及其连接的序列的数据组，在种内及种间遗传距离，并运用邻接法、最大简约法、最小进化法和最大似然法，分别重建系统进化树。
结果：获得了线粒体基因组的 4 个片段，分别为 *COI+tRNA+COⅡ* (F5)、*ATP6+COⅢ*(F7+F8)、*ND1*(F19)和 *lrRNA* (F21)，4 个片段连接组成联合数据，总长度为 3393 bp。所有片段在种内高度保守，最大 *p* 遗传距离为比伦按蚊 F19 片段，为 0.026。基于各独立片段计算的中华按蚊、克莱按蚊和比伦按蚊的成对种间 *p* 距离都很小，甚至没有差别。然而，由联合数据序列计算得到的种间 *p* 遗传距离范围为 0.004（比伦按蚊与克莱按蚊）至 0.089（类中华按蚊与微小按蚊）；同时，赫坎按蚊种团 6 个成员种之间的 *p* 遗传距离均小于 0.029。系统进化树为两个主要分支：其中一支包含所有的按蚊亚属种类，包括赫坎按蚊种团的 6 成员种、黑小按蚊和四斑按蚊；另一支则是塞蚊亚属种类，包括大劣按蚊和微小按蚊。赫坎按蚊种团成员种分为两个姐妹支：((雷氏按蚊，类中华按蚊)，八代按蚊）和（(比伦按蚊，云南中华按蚊)，克莱按蚊)。
结论：本研究基于线粒体基因组片段序列重建的赫坎按蚊种团成员种的亲缘关系，显示分为两个姐妹支，其中的比伦按蚊、中华按蚊和克莱按蚊关系最为密切；更多的分子标记将有助于更好地进行系统进化分析。

Translated from English version into Chinese by Feng Tao

Phylogénie de certains membres du groupe hyrcanus (Diptera : Culicidae) en Chine basée sur des fragments du génome mitochondrial

Hui-Min Zhu, Shu-Han Luo, Man Gao, Feng Tao, Jing-Peng Gao, Han-Ming Chen, Xiang-Yu Li, Heng Peng et Ya-Jun Ma

Résumé

Contexte: Les espèces du groupe *Anopheles hyrcanus* sont largement répandues dans les régions paléarctiques et orientales et certaines d'entre elles sont d’importants vecteurs du paludisme. Il était presque impossible d’identifier les espèces cryptiques du groupe *An. hyrcanus* à partir de leur morphologie seulement. La relation phylogénétique du groupe *An. hyrcanus* était difficile à caractériser.
Méthodes: Cinq membres du groupe *An. hyrcanus* ont été identifiés par le séquençage de l’ADNr ITS2 : *An. yatsushiroensis, An. belenrae, An. kleini, An. lesteri* et *An. sineroides.* Les fragments du génome mitochondrial ont été séquencés et annotés en utilisant le génome mitochondrial de *An. sinensis* comme référence. En se basant sur les quatre segments obtenus et l’assemblage des données de séquençage (*Joint Data*) de ces espèces, et de quatre autres espèces anophèles téléchargées de GenBank, les distances génétiques intraspécifiques et interspécifiques ont été calculées et des arbres phylogénétiques ont été reconstruits à partir des méthodes de *neighbor joining,* de maximum parcimonie, de l’évolution minimum et du maximum de vraisemblance.
Découvertes: Quatre parties du génome mitochondrial, qui étaient des fragments partiels de *COI+tRNA+COⅡ* (F5), *ATP6+COⅢ*(F7+F8), *ND1*(F19) et *lrRNA* (F21), ont été obtenues. Tous les fragments étaient assemblés en une séquence (que l’on désigne comme *Joint Data*), qui avait une

longueur totale de 3393 pb. Toutes les séquences de fragments étaient grandement conservées entre les espèces, avec une distance *p* maximale (0.026) calculée par le F19 de *An. Belenrae*. La distance *p* du pairage interspécifique calculée pour chaque fragment montrait une différence mineure ou inexistante parmi *An. sinensis*, *An. kleini* et *An. belenrae*. Cependant, les distances *p* interspécifiques calculées par la séquence *Joint Data* se situent entre 0.004 (*An. belenrae* vs *An. kleini*) et 0.089 (*An. sineroides* vs *An. minimus*), et les distances *p* des six membres du groupe *An. hyrcanus* étaient toutes de moins de 0.029. L'arbre phylogénétique a montré deux clades majeurs: toutes les espèces du sous-genre *Anopheles* (y compris six membres du groupe *An. hyrcanus*, *An. atroparvus* et *An. quadrimaculatus* A) et du sous-genre *Cellia* (y compris *An. dirus* et *An. minimus*). Le groupe *An. hyrcanus* était divisé en deux groupes : ((*An. lesteri, An. sineroides*) *An. yatsushiroensis*) et ((*An. belenrae, An. sinensis*) *An. kleini*)).
Conclusion: Le groupe *An. Hyrcanus*, dans cette étude, pourrait être divisé en deux groupes, dans l'un desquels *An. belenrae, An. sinensis* et *An. kleini* étaient plus étroitement liés. Plus de marqueurs moléculaires pourraient apporter une plus grande contribution à l'analyse phylogénétique.

Translated from English version into French by Camille Lamoureux, revised by Blandine Mathey, through

TRANSLATORS WITHOUT BORDERS

Филогения некоторых представителей группы Hyrcanus (Diptera: Culicidae) в Китае на основе фрагментов митохондриального генома

Хуэй-Минь Чжу, Шу-Хань Ло, Ман Гао, Фэн Тао, Цзин-Пэн Гао, Хань-Мин Чен, Сян-Ю Ли, Хэн Пэн и Я-Цзюнь Ма

Резюме

Справочная информация: Виды группы *Anopheles hyrcanus* широко распространены в палеарктических и восточных регионах, и некоторые из них являются крупными переносчиками малярии. Загадочные виды группы *An. hyrcanus* было практически невозможно идентифицировать, основываясь только на их морфологии. Филогенетические отношения группы *An. hyrcanus* также были неясны.
Методы: пять членов группы *An. hyrcanus* были идентифицированы методом секвенирования рДНК ITS2 как *An. yatsushiroensis, An. belenrae, An. kleini, An. lesteri* и *An. sineroides.* Фрагменты митохондриального генома секвенировали и аннотировали с использованием митохондриального генома *An. sinensis* в качестве эталона. На основе четырех сегментов и последовательностей совместных данных этих видов и других четырех видов анофелинов, загруженных из GenBank, были рассчитаны как внутривидовые, так и межвидовые генетические расстояния, и филогенетические деревья были реконструированы методами соединения соседей, принципа наибольшей экономии, минимальной эволюции и максимальной вероятности.
Результаты: были получены четыре части митохондриальных геномов, которые были частичными фрагментами *COI + mРНК + COⅡ* (F5), *ATP6 + COⅢ*(F7 + F8), *ND1*(F19) и lrРНК (F21). Все фрагменты были соединены в одну последовательность (называемую «Объединенные

данные») общей длиной 3393 п.н. Все последовательности фрагментов были высоко консервативными в пределах вида, с максимальным расстоянием *p* (0,026), рассчитанным по F19 *An. belenrae*. Попарное межвидовое расстояние *p*, рассчитанное каждым фрагментом, показало незначительное или даже отсутствие различий между *An. sinensis*, *An. kleini* и *An. belenrae*. Тем не менее, межвидовые расстояния p, рассчитанные последовательностью объединенных данных, варьировались от 0,004 (*An. belenrae* против *An. kleini*) до 0,089 (*An. sineroides* против *An. minimus*), а *все расстояния p* для шести членов группы *An. hyrcanus* были менее 0,029. Филогенетическое дерево показало две основных клады (филогенетических ветви): все виды подрода *Anopheles* (включая шесть представителей группы *An. hyrcanus*, *An. atroparvus* и *An. quadrimaculatus* A) и подрод *Cellia* (включая *An. dirus* и *An. minimus*). Группа *An. hyrcanus* была разделена на два кластера: ((*An. lesteri, An. sineroides*) *An. yatsushiroensis*) и ((*An. belenrae, An. sinensis*) *An. kleini*)).
Выводы: группу *An. hyrcanus* в этом исследовании можно разделить на два кластера, в одном из которых *An. belenrae, An. sinensis* и *An. kleini* были наиболее тесно связаны. Большее количество молекулярных маркеров позволило бы провести более тщательный филогенетический анализ.

Translated from English version into Russian by Michael Orlov, revised by Maria Petrenko, through

Filogenia de ciertos miembros del grupo *Hyrcanus (Diptera: Culicidae*), en China, basada en fragmentos del genoma mitocondrial

Hui-Min Zhu, Shu-Han Luo, Man Gao, Feng Tao, Jing-Peng Gao, Han-Ming Chen, Xiang-Yu Li, Heng Peng y Ya-Jun Ma

Resumen
Antecedentes: Las especies del grupo *Anopheles hyrcanus* se distribuyen, ampliamente, en las regiones Paleártica y Oriental y algunas de ellas son los principales vectores de la malaria. Las especies crípticas del grupo *An. hyrcanus* fueron casi imposibles de identificar basándose solo en su morfología. La relación filogenética del grupo *An. hyrcanus* tampoco estaba clara.
Métodos: Cinco miembros del grupo *An. hyrcanus* se identificaron por secuenciación de rDNA ITS2, como *An. yatsushiroensis, An. belenrae, An. kleini, An. lesteriand An. sineroides*. Los fragmentos del genoma mitocondrial se secuenciaron y anotaron, utilizando el genoma de referencia mitocondrial de *An. sinensisas*. Sobre la base de los cuatro segmentos y en las secuencias *Joint Data* de estas especies y otras cuatro especies de anofelina, que se descargaron del *GenBank*, se calcularon distancias genéticas intraespecíficas, así como también interespecíficas y se reconstruyeron los árboles filogenéticos, usando los métodos de unión de adyacentes, máxima parsimonia, evolución mínima y máxima verosimilitud.
Resultados: Se obtuvieron cuatro partes del genoma mitocondrial, los cuales fueron fragmentos parciales *COI+ARNt+COⅡ* (F5), *ATP6+COⅢ* (F7+F8), *ND1* (F19) y*ARNlr* (F21). Todos los fragmentos se conectaron, como una sola secuencia (a la que se hace referencia como *Joint Data*), que tuvo una longitud total de 3393 pb. Todas las secuencias de fragmentos fueron, marcadamente, conservativas, dentro de la misma especie, con una distancia (0,026) *p* máxima calculada por F19 de *An.*

belenrae. La distancia *p* calculada del par inter-específico, por cada fragmento, mostró una diferencia mínima o inexistente entre *An. sinensis*, *An. kleini* y *An. belenrae*. Sin embargo, las distancias *p* interespecíficas calculadas por la secuencia *Joint Data* oscilaron entre 0,004 (*An. belenrae* vs.*An. Kleini*) y 0,089 (*An. sineroides* vs, .*An. minimus*), y las distancias *p* de los seis miembros del grupo *An. hyrcanus* fueron todas menores a 0,029. El árbol filogenético mostró dos claros principios: todos subgéneros *Anopheles* especies (incluyendo seis miembros del grupo *An. hyrcanus*, *An. atroparvus* y *An. quadrimaculatus* A) y subgénero *Cellia* (incluyendo *An. dirus* y *An. minimus*). El grupo *An. hyrcanus* se dividió en dos conjuntos como (*An. lesteri, An. sineroides*) (*An. yatsushiroensis*) y ((*An. belenrae, An. sinensis*) *An. kleini*)).

Conclusiones: El grupo *An. hyrcanus*, en este estudio, se puede dividir en dos conjuntos, en uno de los que *An. belenrae, An. sinensis* y *An. Kleini* estaban más próximamente relacionados. Una mayor cantidad de marcadores moleculares hará una gran contribución al análisis filogenético.

Translated from English version into Spanish by Valeria Esterzon, revised by María Luz Puerta, through

TRANSLATORS
WITHOUT BORDERS
