# Supplementary material for: Phylogeny of certain members of Hyrcanus group (Diptera: Culicidae) in China based on mitochondrial genome fragments
Source: Infect Dis Poverty. 2019 Oct 23;8:91. doi: 10.1186/s40249-019-0601-1 (PMC6806543; doi:10.1186/s40249-019-0601-1)
Supplement: Supplementary file 2 — Additional file 2: Table S1. The mean of nucleotide composition and the numbers of conserved and variable bases of mitochondrial genome fragments in Anopheles hyrcanus group 5 members. [file 40249_2019_601_MOESM2_ESM.docx]

**Table S1** The information of mean nucleotide composition, numbers of conserved and variable bases of mitochondrial genome fragments in 5 *Anopheles hyrcanus* group

| Fragment | T (%) | C (%) | A (%) | G (%) | Total | Conserved | Variable | Parasim-info |
| --- | --- | --- | --- | --- | --- | --- | --- | --- |
| F5 | 39.5 | 14.6 | 34.8 | 11.1 | 893 | 824 | 69 | 48 |
| F7 | 33.4 | 11.3 | 40.0 | 15.3 | 782 | 710 | 72 | 29 |
| F8 | 32.3 | 13.1 | 38.9 | 15.8 | 874 | 810 | 64 | 50 |
| F19 | 28.6 | 14.7 | 47.3 | 9.5 | 583 | 524 | 59 | 36 |
| F21 | 39.0 | 9.4 | 34.7 | 16.8 | 510 | 501 | 9 | 8 |
