# Supplementary material for: Phylogeny of certain members of Hyrcanus group (Diptera: Culicidae) in China based on mitochondrial genome fragments
Source: Infect Dis Poverty. 2019 Oct 23;8:91. doi: 10.1186/s40249-019-0601-1 (PMC6806543; doi:10.1186/s40249-019-0601-1)
Supplement: Supplementary file 3 — Additional file 3: Table S2. The pairwise p distance between Subgenus Cellia and Anopheles species in this study calculated by F5 sequences.) [file 40249_2019_601_MOESM3_ESM.docx]

**Table S2** The pairwise *p* distance between Subgenus *Cellia* and *Anopheles* species in this study calculated by F5 sequences

|  | YAT | BEL | KLE | LES | SINE | SIN | DIR | ATR | QUA |
| --- | --- | --- | --- | --- | --- | --- | --- | --- | --- |
| BEL | 0.034 |  |  |  |  |  |  |  |  |
| KLE | 0.032 | 0.005 |  |  |  |  |  |  |  |
| LES | 0.033 | 0.032 | 0.032 |  |  |  |  |  |  |
| SINE | 0.030 | 0.034 | 0.032 | 0.025 |  |  |  |  |  |
| SIN | 0.034 | 0.007 | 0.009 | 0.036 | 0.036 |  |  |  |  |
| DIR | 0.098 | 0.104 | 0.107 | 0.106 | 0.105 | 0.103 |  |  |  |
| ATR | 0.097 | 0.090 | 0.094 | 0.103 | 0.096 | 0.095 | 0.111 |  |  |
| QUA | 0.090 | 0.092 | 0.092 | 0.090 | 0.088 | 0.095 | 0.099 | 0.076 |  |
| MIN | 0.100 | 0.096 | 0.099 | 0.103 | 0.107 | 0.095 | 0.096 | 0.116 | 0.107 |

YAT: *An. yatsushiroensis*; BEL: *An. belenrae*; KLE: *An. kleini*; LES: *An. lesteri*; SINE: *An. sineroides*; SIN: *An. sinensis*; DIR: *An. dirus A*; ATR: *An. atroparvus*; QUA: *An. quadrimaculatus*; MIN: *An. minimus*.
