# Supplementary material for: Phylogeny of certain members of Hyrcanus group (Diptera: Culicidae) in China based on mitochondrial genome fragments
Source: Infect Dis Poverty. 2019 Oct 23;8:91. doi: 10.1186/s40249-019-0601-1 (PMC6806543; doi:10.1186/s40249-019-0601-1)
Supplement: Supplementary file 4 — Additional file 4: Table S3. The pairwise p distance between Subgenus Cellia and Anopheles species in this study calculated by F7 + F8 sequences. [file 40249_2019_601_MOESM4_ESM.docx]

**Table S3** The pairwise *p* distance between Subgenus *Cellia* and *Anopheles* species in this study calculated by F7+8 sequences

|  | YAT | BEL | KLE | LES | SINE | SIN | DIR | ATR | QUA |
| --- | --- | --- | --- | --- | --- | --- | --- | --- | --- |
| BEL | 0.019 |  |  |  |  |  |  |  |  |
| KLE | 0.018 | 0.001 |  |  |  |  |  |  |  |
| LES | 0.027 | 0.026 | 0.024 |  |  |  |  |  |  |
| SINE | 0.030 | 0.028 | 0.027 | 0.028 |  |  |  |  |  |
| SIN | 0.019 | 0.003 | 0.001 | 0.026 | 0.028 |  |  |  |  |
| DIR | 0.084 | 0.078 | 0.077 | 0.086 | 0.093 | 0.078 |  |  |  |
| ATR | 0.092 | 0.088 | 0.086 | 0.093 | 0.097 | 0.087 | 0.100 |  |  |
| QUA | 0.088 | 0.083 | 0.081 | 0.086 | 0.093 | 0.082 | 0.099 | 0.072 |  |
| MIN | 0.094 | 0.088 | 0.086 | 0.089 | 0.096 | 0.088 | 0.090 | 0.087 | 0.090 |

YAT: *An. yatsushiroensis*; BEL: *An. belenrae*; KLE: *An. kleini*; LES: *An. lesteri*; SINE: *An. sineroides*; SIN: *An. sinensis*; DIR: *An. dirus A*; ATR: *An. atroparvus*; QUA: *An. quadrimaculatus*; MIN: *An. minimus*.
