# Supplementary material for: Phylogeny of certain members of Hyrcanus group (Diptera: Culicidae) in China based on mitochondrial genome fragments
Source: Infect Dis Poverty. 2019 Oct 23;8:91. doi: 10.1186/s40249-019-0601-1 (PMC6806543; doi:10.1186/s40249-019-0601-1)
Supplement: Supplementary file 5 — Additional file 5: Table S4. The pairwise p distance between Subgenus Cellia and Anopheles species in this study calculated by F19 sequences. (DOCX 15 kb) [file 40249_2019_601_MOESM5_ESM.docx]

**Table S4** The pairwise *p* distance between Subgenus *Cellia* and *Anopheles* species in this study calculated by F19 sequences

|  | YAT | BEL | KLE | LES | SINE | SIN | DIR | ATR | QUA |
| --- | --- | --- | --- | --- | --- | --- | --- | --- | --- |
| BEL | 0.015 |  |  |  |  |  |  |  |  |
| KLE | 0.013 | 0.006 |  |  |  |  |  |  |  |
| LES | 0.015 | 0.013 | 0.013 |  |  |  |  |  |  |
| SINE | 0.026 | 0.031 | 0.031 | 0.026 |  |  |  |  |  |
| SIN | 0.015 | 0.007 | 0.007 | 0.017 | 0.035 |  |  |  |  |
| DIR | 0.055 | 0.048 | 0.050 | 0.059 | 0.075 | 0.048 |  |  |  |
| ATR | 0.062 | 0.061 | 0.061 | 0.061 | 0.072 | 0.057 | 0.073 |  |  |
| QUA | 0.059 | 0.050 | 0.051 | 0.050 | 0.070 | 0.048 | 0.073 | 0.064 |  |
| MIN | 0.061 | 0.048 | 0.051 | 0.050 | 0.072 | 0.055 | 0.072 | 0.062 | 0.055 |

YAT: *An. yatsushiroensis*; BEL: *An. belenrae*; KLE: *An. kleini*; LES: *An. lesteri*; SINE: *An. sineroides*; SIN: *An. sinensis*; DIR: *An. dirus A*; ATR: *An. atroparvus*; QUA: *An. quadrimaculatus*; MIN: *An. minimus*.
