# Supplementary material for: Phylogeny of certain members of Hyrcanus group (Diptera: Culicidae) in China based on mitochondrial genome fragments
Source: Infect Dis Poverty. 2019 Oct 23;8:91. doi: 10.1186/s40249-019-0601-1 (PMC6806543; doi:10.1186/s40249-019-0601-1)
Supplement: Supplementary file 6 — Additional file 6: Table S5. The pairwise p distance between Subgenus Cellia and Anopheles species in this study calculated by F21 sequences. [file 40249_2019_601_MOESM6_ESM.docx]

**Table S5** The pairwise *p* distance between Subgenus *Cellia* and *Anopheles* species in this study calculated by F21 sequences

|  | YAT | BEL | KLE | LES | SINE | SIN | DIR | ATR | QUA |
| --- | --- | --- | --- | --- | --- | --- | --- | --- | --- |
| BEL | 0.002 |  |  |  |  |  |  |  |  |
| KLE | 0.002 | 0.000 |  |  |  |  |  |  |  |
| LES | 0.012 | 0.014 | 0.014 |  |  |  |  |  |  |
| SINE | 0.002 | 0.004 | 0.004 | 0.010 |  |  |  |  |  |
| SIN | 0.004 | 0.002 | 0.002 | 0.016 | 0.006 |  |  |  |  |
| DIR | 0.036 | 0.038 | 0.038 | 0.042 | 0.034 | 0.036 |  |  |  |
| ATR | 0.024 | 0.022 | 0.022 | 0.030 | 0.022 | 0.020 | 0.040 |  |  |
| QUA | 0.024 | 0.022 | 0.022 | 0.032 | 0.026 | 0.020 | 0.040 | 0.012 |  |
| MIN | 0.048 | 0.050 | 0.050 | 0.058 | 0.050 | 0.048 | 0.060 | 0.052 | 0.044 |

YAT: *An. yatsushiroensis*; BEL: *An. belenrae*; KLE: *An. kleini*; LES: *An. lesteri*; SINE: *An. sineroides*; SIN: *An. sinensis*; DIR: *An. dirus A*; ATR: *An. atroparvus*; QUA: *An. quadrimaculatus*; MIN: *An. minimus*.
