# Supplementary figures and images for: Phylogeny of certain members of Hyrcanus group (Diptera: Culicidae) in China based on mitochondrial genome fragments
Source: Infect Dis Poverty. 2019 Oct 23;8:91. doi: 10.1186/s40249-019-0601-1 (PMC6806543; doi:10.1186/s40249-019-0601-1)

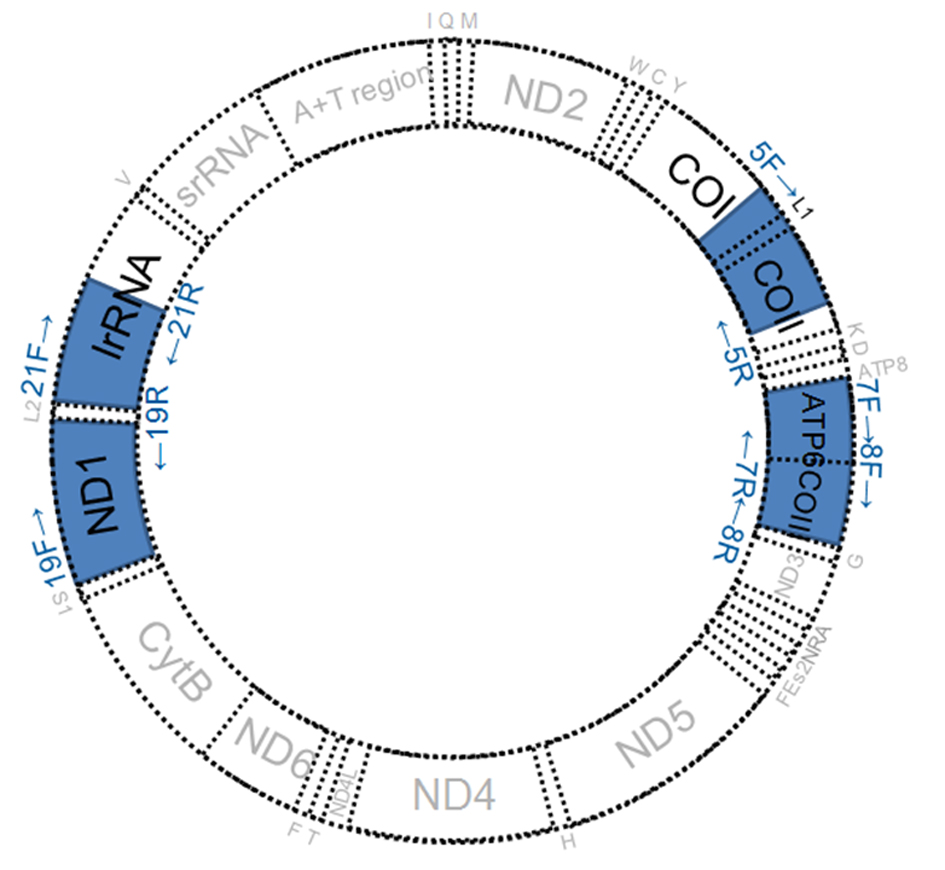

Supplement: Supplementary file 7 — Additional file 7: Figure S1. Primers for amplifying mitochondrial genome fragments of Anopheles. [file 40249_2019_601_MOESM7_ESM.jpg]

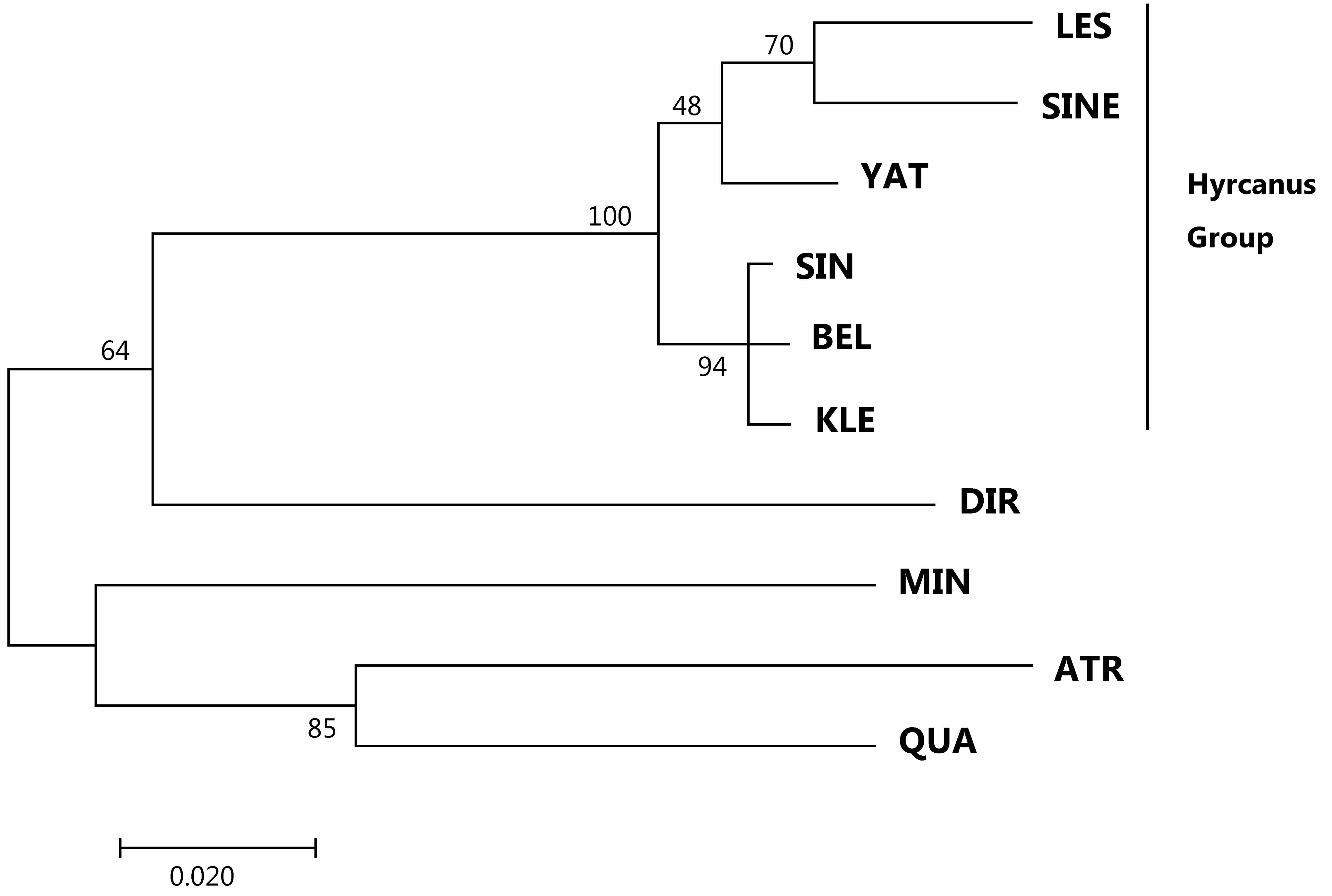

Supplement: Supplementary file 8 — Additional file 8: Figure S2. The phylogenetic ML tree reconstructed based on F7 + F8 fragment of mitochondrial genome. YAT: An. yatsushiroensis; BEL: An. belenrae; KLE: An. kleini; LES: An. lesteri; SINE: An. sineroides; SIN: An. sinensis; DIR: An. dirus A; ATR: An. atroparvus; QUA: An. quadrimaculatus; MIN: An. minimus. The numbers on the clades denote the bootstrap confidence values. [file 40249_2019_601_MOESM8_ESM.tif]
